# Changes in Prosocial Behaviors Among Children With Behavioral Problems Following Incredible Years Parenting Program

**DOI:** 10.3389/fpsyg.2022.847722

**Published:** 2022-05-04

**Authors:** Ruziana Masiran, Normala Ibrahim, Hamidin Awang, Poh Ying Lim

**Affiliations:** ^1^Department of Psychiatry, Faculty of Medicine and Health Sciences, Universiti Putra Malaysia, Serdang, Malaysia; ^2^Department of Psychiatry, Hospital Pengajar Universiti Putra Malaysia (HPUPM), Serdang, Malaysia; ^3^Department of Community Health, Faculty of Medicine and Health Sciences, Universiti Putra Malaysia, Serdang, Malaysia

**Keywords:** behavior problem, children, incredible years, intervention, Malaysia, parenting, parenting program, prosocial behavior

## Abstract

Parenting programs often train parents in improving their parenting practices and parent-child relationship to reduce behavioral problems in children. However, the children’s prosocial behaviors are less examined as an intervention outcome in these programs. This study aimed to evaluate the effectiveness of the Incredible Years parenting program (IYPP) for Malaysian parents of school-going children and its sustainability in improving the children’s prosocial behaviors. This randomized controlled study involved pre- and post-intervention assessments at 2 and 14 weeks. Mothers of children aged 6-12 years (*n* = 70) recruited through the pediatrics and the child and adolescent psychiatric clinics were randomly assigned to the parenting program or a waitlist control condition. The mothers rated their children’s prosocial behaviors using a self-administered questionnaire. The program ran two to three hours weekly for 14 weeks. Several modifications were made to the program to accommodate public health control during the pandemic. Children in the intervention group showed a notable but non-significant increase in prosocial skills. However, subsequent score decline at follow-up may suggest a lack of evidence that the program is potentially effective in improving prosocial behaviors among school children who are at risk of or already having behavioral problems.

## Introduction

Social competence is a major childhood developmental task that needs to be accomplished as it promotes positive and supportive future interpersonal relationships (Ma, 2012). Children who are more socially competent are more likely to engage in prosocial behaviors, a set of social competence measures (Rydell et al., 1997; Ma, 2012). Prosocial behaviors are behaviors that are voluntarily and deliberately done to benefit others ‘for which the motive is unspecified, unknown, or not altruistic’, including helping people and sharing objects (pp. 6) (Eisenberg, 1982). The earliest definition and concept of prosocial behavior was introduced by Nancy Eisenberg, who differentiated prosocial behaviors and altruistic behaviors based on the underlying motives (pp. 3) (Eisenberg and Mussen, 1989). Eisenberg (1982) had argued that different moral reasoning, such as compensation, recognition, or simply caring about others, may justify prosocial acts (pp. 234). While the development of these behaviors is rooted in cognitive, social learning, and biological theories, cultural variations may play a role in the nurturance of these behaviors in children (Hammond et al., 2015). Therefore, nurturing prosocial behaviors in children is valuable for a child’s social competence (Ma, 2012) and their social well-being in adulthood (Biglan et al., 2012; Vergunst et al., 2019). Besides, prosocial behaviors are positively associated with academic outcomes (Carlo et al., 2018) and parenting practices (Pastorelli et al., 2016), and also transcend cultures. In an analysis performed in eight countries (Colombia, Italy, Jordan, Kenya, the Philippines, Sweden, Thailand, and the United States), Pastorelli et al. (2016) demonstrated that prosocial behaviors in late childhood enhance positive maternal parenting. On the other hand, prosocial behaviors are negatively associated with children’s internalizing and externalizing behavioral problems, high-risk sexual behaviors, and substance use (Padilla-Walker et al., 2015; Flouri and Sarmadi, 2016; Bagán et al., 2019; Memmott-Elison et al., 2020).

Positive parenting, which comprised of parental warmth and support (Llorca et al., 2017), parental involvement (Ogunboyede and Agokei, 2016), as well as supportive emotion socialization (Acar-Bayraktar et al., 2019), promotes prosociality in children (Llorca et al., 2017). In an experiment by Acar-Bayraktar et al. (2019), positive maternal reactions were found to be positively associated with prosocial behaviors in children. This finding is in agreement with the literature, which showed that parental practices of empathic responding (Eisenberg et al., 2010; Van der Graaff et al., 2018), providing sympathetic expression (Eisenberg et al., 2010), and modeling good behaviors (Schuhmacher et al., 2019), promote prosocial behaviors. Furthermore, Baez et al. (2017) and Van der Graaff et al. (2018) highlighted that moral emotions foster prosocial behaviors. According to Haidt (2003), moral emotions are ‘those emotions that are linked to the interests or welfare either of society as a whole or at least of persons other than the judge or agent’ (p. 853). Therefore, it was concluded that prosocial moral reasoning lead to prosocial actions (Carlo et al., 2010). Furthermore, girls have been found to be more prosocial than boys (Altay and Gure, 2012). According to Orte et al. (2015), positive parenting is predictive of prosocial behaviors in boys, while girls’ prosocial behaviors are influenced by the parent-children relationship. Another important parenting factor is parenting style (Malonda et al., 2019). Children of authoritative parents showed more prosocial behaviors compared to children whose parents were more demanding, less involved (Carlo et al., 2018), or permissive (Altay and Gure, 2012). This is in line with a recent study by Wong et al. (2021) that documented a positive association between authoritative parenting and prosocial behaviors. Also, it was argued that maternal parenting plays a larger role in encouraging prosocial behaviors than paternal parenting (Bagán et al., 2019).

Evidence showed that behavioral strategies are employed in the majority of interventions that promote prosocial skills (Laguna et al., 2020). Based on previous data, there was a significant negative correlation between behavioral problems and social competence (*r* = −0.42, *p* < 0.001) (Hukkelberg et al., 2019), which indicated that interventions aimed at behavioral problems are expected to increase social competence. Correspondingly, parenting programs have moved forward from eliminating disruptive behaviors to increasing prosocial skills in children (Shaffer et al., 2001). In these programs, parents are commonly trained to teach children to share and get along with peers (Kaminski et al., 2008). One such program is the Incredible Years parenting program (IYPP) (Webster-Stratton and Reid, 2011), a widely-researched and effective series of parent training programs that focuses on enhancing positive parent-child interactions. In early research that utilized this program, prosocial behaviors displayed immediate increment after program completion and six months afterward (Patterson et al., 2002). Consistent with that, a meta-analysis by Menting et al. (2013) supported its use to improve children’s prosocial behaviors (*d* = 0.23). Subsequent research on the efficacy of the IYPP among preschoolers showed that enhanced prosocial behaviors occurred in tandem with improved parenting (Seabra-Santos et al., 2016). Nevertheless, the impact of this program on prosocial behaviors is still rarely been evaluated (Mingebach et al., 2018). This current study aimed to evaluate the effectiveness of the IYPP to improve prosocial behaviors among Malaysian school-going children with behavioral problems. It was hypothesized that the program would increase the scores of prosocial behaviors of these children.

## Materials and Methods

The study used an experimental randomized controlled between-group design. Pre- and post-intervention assessments were conducted, with post-intervention assessments at 2 and 14 weeks.

### Participants

Children were referred by their treating pediatricians and psychiatrists from the pediatrics and child and adolescent psychiatric (CAP) clinics of Kajang, Kuala Lumpur, and Selayang Hospitals in Malaysia. Children aged 6-12 years old were eligible if they had clinical levels of behavioral problems, indicated by their mothers’ ratings on the Strengths and Difficulties Questionnaire (SDQ) total difficulty scores of ≥15, i.e., the 80th percentile (borderline and abnormal levels) (Gomez and Suhaimi, 2013). Other inclusion criteria include the mothers must have lived with the children for at least six months, agreed to participate, and were not attending other parenting programs during the study period. Children were excluded from the study if they had a formal diagnosis of autism spectrum, severe neurological, or developmental disorder.

### Sample Size Calculation

The formula for continuous response variables in hypothesis testing for two means by Lemeshow et al. (1990) was used to calculate the sample size. To determine the effectiveness of the IYPP, the Type 1 error (α) was set to be 0.05, to give a statistical power of 80% in order to detect an effect size of 0.50 (medium) in the total behavioral problem, among participants of the IYPP compared to the control group, based on the findings by Morpeth et al. (2017). The minimum sample size required was a total of 34 parents; 17 for the intervention group and 17 for the control group. Nonetheless, based on their systematic review on behavioral parent training, Chacko et al. (2016) found a combined dropout rate of 51%. Therefore, in order to achieve a medium effect size, and allowing for a dropout rate of 50%, the total sample size will be [34 × 1/(1−0.50)] = 69.4, with 35 participants for each group.

### Procedures

**FIGURE 1:**
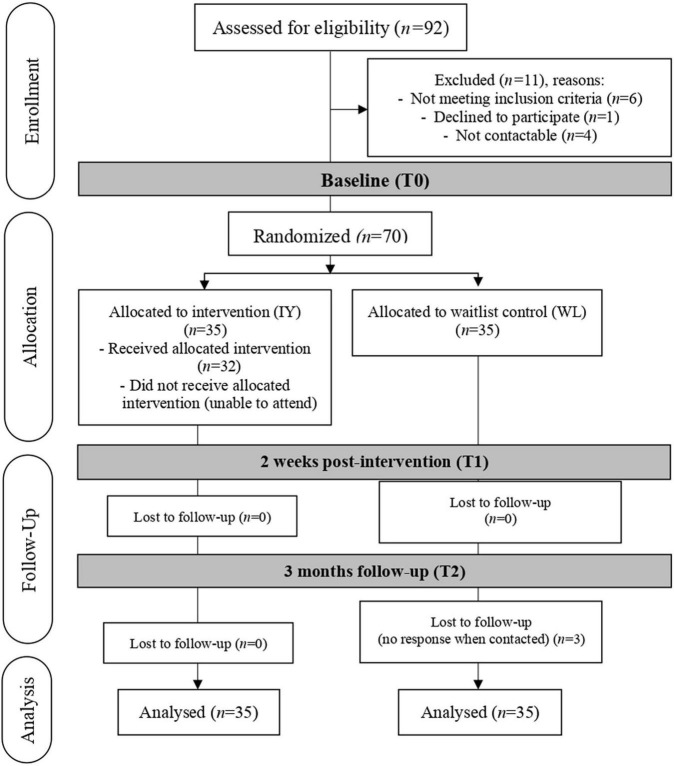
The flow of study. Adapted from “CONSORT 2010 statement: Updated guidelines for reporting parallel group randomized trials”, by Schulz et al., 2010. CC BY 2.0.

Ninety two mother child-dyads were referred by clinicians. After assessing for eligibility, there were 81 mother-child dyads in the sampling frame, of which simple random sampling was performed to select 70 mother-child dyads. After the baseline assessment, mother-child dyads were randomly allocated to the IYPP group (IY; *n* = 35) or the waitlist control group (WL; *n* = 35) (Figure 1). A research assistant who was not involved in data collection or intervention randomly assigned participants using an online random sequence generator on a 1:1 basis. Data were collected by research assistants who were blinded to the participant’s group assignment, and mothers were asked not to reveal whether or not they had attended a group. The data collection was done three times and scheduled on different days for the intervention and control groups. Participating mothers were also given non-overlapping follow-up dates with their children’s treating doctors to prevent treatment contamination.

### Ethical Considerations

This study was authorized by the Medical Research and Ethics Committee of the Ministry of Health, Malaysia and the Ethics Committee for Research Involving Human Subject of Universiti Putra Malaysia (JKEUPM). Mothers who agreed to participate were given an information packet containing a cover letter describing the study and an informed consent form. Eligible parent-child dyads who gave their informed consents were enrolled and included into the participant list. As a small compensation for their participation in the research and completing the questionnaires at baseline, post-intervention, and follow-up, mothers received RM10 cash along with travelling cost reimbursement.

### Measures

#### Sociodemographic Data

The sociodemographic details of participants contained the following information:

The sociodemographic factors included in the analysis were the parental factors: age, ethnicity, marital status, number of children, education level, income; child factors: age, gender, and ethnicity. presence of attention-deficit/hyperactivity disorder (ADHD), and prescription of stimulants.

#### Clinical Data

The clinical profile of participants contained the following information:

(i) Name referring hospital (where the child was being followed-up).
(ii) Presence of attention deficit/hyperactivity disorder (ADHD) diagnosis.
(iii) Prescription of stimulant.

#### Measures of Child Behavior

The Malay version of parent-rated 25-item Strengths and Difficulties Questionnaire (SDQ) (Goodman, 1997) was used to assess the emotional and behavioral symptoms in children and adolescents aged 4-17 years. Scores from four difficulties subscales (emotional problems, conduct problems, hyperactivity symptoms, and peer problems) were combined to generate total difficulties score between 0 and 40. The prosocial subscale (SDQ-PRO) was grouped separately, with a higher score indicated more prosocial behaviors. Children were then categorized into normal, borderline, or abnormal bands based on the sum of SDQ scores, but it was previously noted that the cut-off scores may vary by culture (Goodman, 1997). Accordingly, the present study used the Malaysian cut-off score for total difficulties: 0-14 = normal, 15-17 = borderline, and 18-40 = abnormal (Gomez and Suhaimi, 2013). The SDQ has high internal consistency, test-retest reliability, and strong criterion validity for predicting psychological disorders (Goodman, 1997). The Malay parent-rated SDQ used in this study show acceptable internal consistencies with Cronbach α values above 0.70 for all SDQ scales except for conduct and peer problems.

### Intervention

#### Program Background

The IYPP equips parents with parenting skills by employing role-plays, video modeling, coaching, self-reflection, individual goal setting, assignments, and experiential learning (The Incredible Years^®^, 2013). The IYPP School Age Basic, which aimed at children aged 6-12 years old, was selected as the program version for the present study. The session topics in the Incredible Years^®^ manual were delivered comprehensively to ensure fidelity and to achieve the intended program outcomes. Two additional sessions were added on top of the 12 intended sessions due to the higher risk of internalizing and externalizing disorders in the participating children and the requirement for intra-session translation (Webster-Stratton, 2009). For each session, 11-12 mothers attended and were divided into three separate parent groups. Each session lasted between two and three hours. Participants who missed a session would be contacted via phone and invited to participate in a make-up session any day before the next session. This make-up session was conducted in the same venue with the intervention program, and could be one-on-one or small group sessions, depending on attendance. In these sessions, shorter versions of the previous week’s group training were done, which lasted for 30-45 min. However, a make-up session was not considered as a session attended. The parent training sessions began in the final week of February and ended in the first week of September 2020. Due to the COVID-19 pandemic, the national lockdown was announced in mid-March 2020.

#### Fidelity Measures

The sessions for all three groups were facilitated by a parenting group leader and a co-facilitator. The group leader had completed a compulsory three-day accredited workshop and was in the process of being certified. Both of them had vast experience in child psychology/psychiatry and have been working with families. Sessions were videotaped for weekly self-review and regular peer supervision.

#### Adaptation to the Program

During the training, minimal adaptation was done by providing participants with culturally relevant examples related to the video and the role-plays. However, as the coronavirus pandemic led to the first national lockdown (Shanmugam et al., 2020) right after three parent training sessions, some changes to the program’s schedule and delivery were made. The subsequent training sessions resumed after three months of lockdown in the previous, face-to-face format. Additionally, the facilitators and participants were kept in contact through text messages via smartphones. A weekly text message served as reminders for mothers to continue practicing the parenting skills learned during the first three parent training sessions. Each weekly reminder came with photos of the notes taken during these sessions. As mentioned, another adaptation was the three make-up classes conducted through online conferencing for mothers who missed any of the sessions.

### Statistical Analysis

Statistical analysis was performed using the Statistical Package for Social Sciences (SPPS) Software Version 25.0. The two-tailed statistical significance was set at *p* < 0.05 and expressed as a 95% confidence interval, and the power fixed at 80%. Missing values were replaced with the last-observation-carried-forward method. The sociodemographic and clinical factors included in the analysis were the parental factors: age, ethnicity, marital status, education level, number of children, total family income, motivation level, and parenting self-efficacy; child factors: age, birth order, gender, education setting, presence of attention-deficit/hyperactivity disorder (ADHD), and prescription of stimulant.

Baseline comparisons between the IY and WL groups were performed using the independent *t*-test or Mann-Whitney U tests for continuous outcome variables and the Chi-square test for categorical outcome variables. Fisher’s exact test was used if the expected value in each cell was less than five. To evaluate the effectiveness of the intervention, generalized estimating equations (GEE) analysis with the intention-to-treat approach was applied. The robust estimator was used for the covariance matrix and the working correlation matrix employed was unstructured. The outcomes of prosocial behaviors (SDQ-PRO) were regarded as the dependent variable. Cohen’s d effect sizes were calculated by dividing the overall mean difference between the groups (expressed as regression coefficient) with the overall standard deviation of the observed data. Effect sizes ≥ 0.8 were considered large, between 0.5 and 0.8 were considered moderate, and between 0.2 and 0.5 were considered small.

## Results

### Sample Characteristics at Baseline

Seventy mother-child dyads participated in the study, and 32 participants attended at least one of the 14 sessions (91.4%). The dropout rate was 15.6% (*n* = 5). The sociodemographic characteristics of IY and WL groups are shown in Table 1. The baseline prosocial behavior scores (SDQ-PRO) between the IY and WL groups showed no significant mean difference [6.26 ± 2.49 vs. 6.57 ± 2.63, t(68) = −0.51, *p* < 0.609]. There was a significant difference in mother’s education level between the intervention and control groups (*x*^2^(1) = 4.64, *p* = 0.031). The equations should be inserted in editable format from the equation editor.

**TABLE 1:** Baseline comparison of sociodemographic and clinical characteristics between IY and WL groups.

Variables	IY (*n* = 35)	WL (*n* = 35)	*x^2/^t*	*df*	*p*
	*n* (%)/ mean ± sd	*n* (%)/ mean ± sd			
**Mothers**					
*Ethnicity*					
Malay	32 (91.4)	31 (88.6)	1.00*^a^*		
Non-Malay	3 (8.6)	4 (11.4)			
*Marital status*					
Married	32 (91.4)	32 (91.4)	1.00*^a^*		
Divorced	3 (8.6)	3 (8.6)			
*Education level*					
Secondary	14 (40)	23 (65.7)	4.64	1	0.031*
Tertiary and above	21 (60.0)	12 (34.3)			
*Total monthly family income*					
<RM 3000	15 (42.9)	12 (34.3)	0.54	1	0.461
≥RM 3000	20 (57.1)	23 (65.7)			
Age	37.60 ± 5.01	38.31 ± 5.96	−0.54^*t*^	68	0.589
Number of children	3.31 ± 1.16	3.60 ± 1.48	−0.90^*t*^	68	0.371
Parent motivation	109.43 ± 12.04	106.29 ± 12.89	1.05^*t*^	68	0.296
**Children**					
*Gender*					
Male	25 (71.4)	21 (60.0)	1.01	1	0.314
Female	10 (28.6)	14 (40.0)			
*Ethnicity*					
Malay	32 (91.4)	31 (88.6)	1.00*^a^*		
Non-Malay	3 (8.6)	4 (11.4)			
Referring hospital					
Kajang	25 (71.4)	25 (71.4)	1.00*^a^*		
Kuala Lumpur/Selayang	10 (28.6)	10 (28.6)			
*Presence of ADHD*					
Yes	10 (28.6)	13 (37.1)	0.58	1	0.445
No	25 (71.4)	22 (62.9)			
*Prescription of stimulant*					
Yes	6 (17.1)	9 (25.7)	0.76	1	0.832
No	29 (82.9)	26 (74.3)			
Age	8.51 ± 1.74	8.54 ± 1.90	−0.07^*t*^	68	0.948

^a^Fisher’s exact test; x ^2^: Chi-square statistics; t: Independent t-test; * p < 0.05.

### Program Attendance and Dropouts

Attendance of at least one session was calculated as the number of attended sessions. 91.4% of the 35 participants in the intervention group, attended at least one session, and eight (25.0%) of them never missed any session. Five participants (15.6%) were categorized as dropouts, meaning they did not return to the program after missing a session.

### Program Effects on Prosocial Behaviors

**FIGURE 2:**
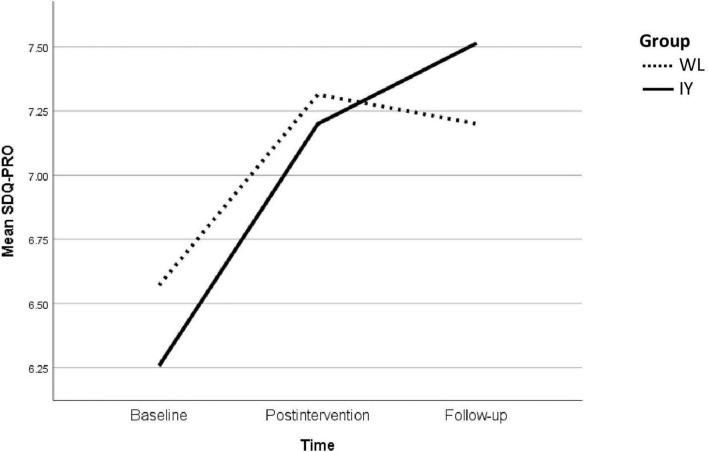
Changes in the prosocial behavior scores (SDQ-PRO) among children in the intervention (IY) and control (WL) group across time.

Table 2 displays the GEE analysis on the effectiveness of the IYPP in improving the SDQ-PRO in children with emotional and/or behavioral problems (EBP) after controlling the sociodemographic and clinical characteristics and number of attended sessions. The IY group produced 0.20 and 0.63 SDQ-PRO points higher than the WL group at 2 weeks post-intervention and 3 months follow-up, respectively (B = 0.20, 95% CI: −0.67, 1.07, *p* = 0.652; B = 0.63, 95% CI: −0.38, 1.64, *p* = 0.0221). A one-point increase in baseline prosocial behaviors was associated with 0.68-point increase in SDQ-PRO (B = 0.68, 95% CI: 0.63, 0.74, *p* < 0.001). The changes in the SDQ-PRO for the IY and WL groups are shown in Figure 2.

**TABLE 2:** Prosocial behavior scores (SDQ-PRO) at baseline, post-intervention, and follow-up between groups.

Variables	Crude B	SE	95% CI	*p*	*d*	Adj. B	SE	95% CI	*p*	*d*
**Group**										
Intervention	−0.16	0.18	−0.52, 0.20	0.379		−0.15	0.20	−0.54, 0.24	0.445	
Control	Ref					Ref				
**Time**										
Follow-up	0.63	0.35	−0.05, 1.31	0.071		0.63	0.35	−0.05, 1.31	0.071	
Post-intervention	0.74	0.29	0.18, 1.31	0.010*		0.74	0.29	0.18, 1.31	0.010*	
Baseline	Ref					Ref				
**Group x Time**										
Intervention x Follow-up	0.63	0.51	−0.38, 1.64	0.221		0.63	0.51	−0.38, 1.64	0.221	
Intervention x Post-intervention	0.20	0.44	−0.67, 1.07	0.652		0.20	0.44	−0.67, 1.07	0.652	
Control x Baseline	Ref					Ref				
Baseline prosocial behaviors	0.70	0.03	0.64, 0.76	< 0.001**		0.68	0.03	0.63, 0.74	< 0.001**	
**Mother’s education level**										
Tertiary and above	0.26	0.16	−0.06, 0.58	0.108		0.36	0.15	0.06, 0.65	0.017*	
Secondary	Ref					Ref				
Child’s gender	−0.40	0.17	−0.72, −0.07	0.016*		−0.34	0.16	−0.66, −0.03	0.035*	
Child’s age	0.10	0.04	0.02, 0.17	0.013*		0.08	0.04	0.01, 0.15	0.033*	
Intercept						1.48	0.41			

Model fit (QIC): 400.919 (backward variable selection method); B: regression coefficient from GEE analysis; positive values indicate positive effects, and vice versa. Adj: adjusted; SE: standard error; CI: 95% confidence interval; ref: reference; d: overall effect size represented as Cohen’s d^;^ * p < 0.05; ** p < 0.001.

## Discussion

This study aimed to determine the effectiveness of the Incredible Years parent training program in improving prosocial behaviors among Malaysian primary school children with clinically significant behavioral problems. This was performed by comparing the prosocial subscale scores between the intervention and waitlist control groups over some time. Overall, the results revealed a non-significant increase in the children’s prosocial behaviors. Nonetheless, this study contributes to the existing evidence on the program’s impact on children’s prosocial behaviors. Through the implementation of an evidence-based parenting program with high fidelity, the study demonstrates that an evidence-based, foreign parenting program with minimal adaptation could be promising even in countries with limited resources.

The primary result indicated that the prosocial behavior scores of the intervention group increased shortly after program completion and enhanced further three months later, long after the mothers were trained. These results support the meta-analytic finding that parenting programs that emphasize positive reinforcement techniques and natural/logical consequences are effective in improving children’s behaviors (Leijten et al., 2019). Although the outcome of this study did not reach statistical significance, this finding may reflect the importance of positive parenting within the intervention of choice. This is in line with the goals of the IYPP to promote parenting competence by increasing positive parenting and parent-child relationships (Webster-Stratton et al., 2016). Additionally, the underlying behavioral approach highlights the importance of appreciating children’s prosocial behaviors through positive reinforcement measures, such as praising and giving rewards.

The lack of significant increment in parent-rated prosocial behavior scores at post-intervention in this current study is contrary to that of Seabra-Santos et al. (2016), who found that IYPP significantly improved child behaviors, measured through rating and direct observation. This inconsistency may be due to using only the parental self-report method without complementation of observation measurement in the present study. This could prove disadvantageous as the parent-report measure is a less objective assessment of a child’s behavior, and it does not provide a more holistic picture of the study outcome as compared to outcome observations. Despite its high cost (Hawes and Dadds, 2006), direct behavioral observation provides an objective examination of the relationship between a child’s prosocial behaviors and the parenting they received. On the other hand, child-rated prosocial behaviors might be more relevant when children are cognitively advanced enough to report on their own behavior (Pastorelli et al., 2016).

Apart from that, parental reports could be biased due to various parental factors. In particular, there was a high level of parental burnout among Malaysian parents (Manja et al., 2020) and depression, anxiety, and stress among adults (Perveen et al., 2020) in the wake of the pandemic. The lockdown was slightly relaxed between the second and third data collection but was retightened when the number of positive COVID-19 cases rose. During this period, the schooling system went from in-person learning to online and back to in-person again. It has been reported that home-based schooling brought a great deal of stress to parents, and the pre-existing behavioral problems in children exacerbated parenting stress (Meltzer et al., 2011). Moreover, there was also a spill-over effect from parents’ lockdown-induced stress or anxiety to children’s emotional and behavioral problems during the lockdown (Spinelli et al., 2020; Dubois-Comtois et al., 2021). This proves that children are equally vulnerable and easily influenced by the mental state of their parents (Imran et al., 2020). Also, young children tend to throw tantrums and project more emotional or behavioral problems during the pandemic (Singh et al., 2020). As a result of this parent and child emotional turmoil, children’s prosocial behaviors may be less than ideal and parents may not be able to give an objective assessment of their children during this period.

Looking at the prosocial behavior scores in the control group, the children exhibited higher scores at post-intervention than baseline, but the scores declined lower three months later. The initial climb could be explained by the fact that the mothers in the waitlist control group were still able to continue their children’s follow-up with their pediatricians or psychiatrists. Consequently, the children would still receive a form of therapy, which could contribute to the enhancement of prosocial behaviors above the baseline. Furthermore, the possibility of compensatory rivalry among the wait-listed mothers should not be underestimated. The mothers may seek for any parenting input to supplement their parenting practices and indirectly improved their children’s behavior. The decline of prosocial scores at 14 weeks post-intervention could be attributed to the lack of parent training on behavioral approaches, leading to the decrease in good behaviors.

In general, literature had strongly suggested the effectiveness of the IYPP in improving children’s behaviors with small to large effect sizes (Javier et al., 2016; Seabra-Santos et al., 2016; Morpeth et al., 2017; Murray et al., 2017; Weeland et al., 2017; Leckey et al., 2018; Leijten et al., 2018a; Gardner et al., 2019; Karjalainen et al., 2019; van Aar et al., 2019). Except for Weeland et al. (2017), who studied the program’s effect on 4-8 year-olds, most of the recent IYPP studies focused on preschool children of a much younger age. One of the few published IYPP studies on school-aged children was conducted by Javier et al. (2016) on Asian children living in the United States. The researchers found a medium effect size in improving the total behavioral problems of children aged 6-12 years old at post-intervention. Another study that included both preschool and school-aged children indicated a significant and large effect in the reduction of the intensity and hyperactivity symptoms of the behavioral problem (ds = 1.51 and 0.81, respectively) (Högström et al., 2017).

In most studies, the child behavioral outcomes were measured in the form of total difficulties or using the internalizing/externalizing behavioral dimensions. Total difficulties are generated by summing scores from all the scales except the prosocial. The internalizing dimensions consist of emotional and peer problem subscales while externalizing dimensions include conduct problem and hyperactivity subscales in the SDQ (Goodman et al., 2010). Similarly, published studies often documented evidence on the positive effects of IYPP on disruptive behaviors (van Aar et al., 2019), such as to conduct problems and hyperactivity that were measured with the Child Behavior Checklist (CBCL) (Javier et al., 2016), Eyberg Child Behavior Inventory (ECBI) (Högström et al., 2017; Weeland et al., 2017), and SDQ (Seabra-Santos et al., 2016; Morpeth et al., 2017). However, only a minority of studies showed that children with higher levels of emotional problems benefited more from IYPP (Leijten et al., 2018a), while others reported no effect (Gardner et al., 2017; Morpeth et al., 2017; Leijten et al., 2018b). This suggests that prosocial behaviors are not commonly measured as a parent training outcome.

The predominant effect of the IYPP on externalizing behavior rather than internalizing shows that the two behavioral dimensions are most likely different concepts. Similar to prosocial skills, internalizing behaviors are related to social processing, problem-solving, and coping strategies (Caputi and Schoenborn, 2018), which are distinct from the behavioral and coercion models underpinning many behavior-based parenting interventions. Like most established parenting programs, the IYPP emphasizes the importance of coercive parenting in interactions with children, enabling it to have a longer effect on externalizing behavior (van Aar et al., 2017). The program’s schedule was designed with the first five sessions focusing on providing positive attention, social skills, and emotion and giving persistent coaching and encouragement. These are the parts where mothers were trained to promote prosocial behaviors in their children. The subsequent sessions were heavily centered on strategies to reduce disruptive/externalizing behaviors, including Clear Limit Setting, Ignoring Misbehavior, Time Out to Calm Down-Discipline Strategies for Excessive Child Disobedience & Hitting or Destructive Behaviors, and Natural & Logical Consequences. This sequence of topics helps them to memorize and be more inclined to use disciplinary strategies rather than the strategies aiming to improve parent-child interaction, the child’s social skills, and emotional regulations. Hence, the strategies to deal with the disruptive behaviors of children would be practiced more.

### Limitations of Study

Due to the small sample size in the present study, the statistical power to detect the difference between the intervention and control groups may have been limited, resulting in false-negative or type II errors. Therefore, future studies must utilize a larger sample to obtain more reliable results with greater precision and power. Another important limitation is the issue of internal validity, as the program had to be temporarily halted for three months after the third session because of the national lockdown. As a result, the internal validity of the study could have been attenuated by the maturation effect. Therefore, various control measures were taken, such as mothers were reminded about the principles and techniques they have learned through text messaging, and the training materials from the first three sessions were revisited when the sessions resumed. In addition, despite the presumed usual contact with the children’s treating doctors for the control group, it was realized that additional details on this ‘usual contact’ should have been obtained. During the usual clinic follow-ups, doctors may have inadvertently imparted parenting advice and become the source of treatment contamination. Parents could similarly be exposed to several psychoeducational approaches concerning parenting during the pandemic through mass and social media. Finally, the sole use of parental self-report measures could cause bias. Direct behavioral observation is a better aid in the systematic analysis of the relationship between the child’s behaviors and the family environment. Nonetheless, despite being the gold standard for the assessment of parenting practices (Zahidi et al., 2019), observational methods are seldom applied due to their high cost (Hawes and Dadds, 2006).

### Implications and Recommendations

This study presents a preliminary indication of the potential effectiveness of the IYPP in promoting prosocial behaviors among school children who are at high risk of or already have behavioral problems. In the clinical setting, it would be beneficial to identify children who would benefit the most from the program. Moreover, an analysis of the program’s effect on each SDQ subscale may reveal whether the program would improve a child’s disruptive behaviors, emotions, peer problems, or prosocial behaviors. Lastly, Malaysian clinicians and policymakers should work together to make the provision of professional training and organizational support for staff possible, so that the IYPP may also benefit families in the community settings.

## Conclusion

The Incredible Years School Age Basic parenting program demonstrated a non-significant increase in the prosocial behavior scores among Malaysian primary school-going children with borderline and abnormal levels of behavioral problems. Subsequent score decline at follow-up suggests a lack of evidence that the program is potentially effective in improving prosocial behaviors.

## Data Availability Statement

The original contributions presented in the study are included in the article/supplementary material, further inquiries can be directed to the corresponding author.

## Ethics Statement

The studies involving human participants were reviewed and approved by Medical Research and Ethics Committee of the Ministry of Health, Malaysia and the Ethics Committee for Research Involving Human Subject of Universiti Putra Malaysia (JKEUPM). Written informed consent to participate in this study was provided by the participants.

## Author Contributions

RM, NI, and HA contributed to the research design and implementation. RM and PYL were involved in data analysis and interpretation. RM and NI participated in the preparation of the manuscript. All authors contributed to the article and approved the submitted version.

## Conflict of Interest

The authors declare that the research was conducted in the absence of any commercial or financial relationships that could be construed as a potential conflict of interest.

## Publisher’s Note

All claims expressed in this article are solely those of the authors and do not necessarily represent those of their affiliated organizations, or those of the publisher, the editors and the reviewers. Any product that may be evaluated in this article, or claim that may be made by its manufacturer, is not guaranteed or endorsed by the publisher.
